# Analytical Modeling for Identification of the Machine Code Architecture of Cyberphysical Devices in Smart Homes

**DOI:** 10.3390/s22031017

**Published:** 2022-01-28

**Authors:** Igor Kotenko, Konstantin Izrailov, Mikhail Buinevich

**Affiliations:** 1 Computer Security Problems Laboratory, St. Petersburg Federal Research Center of the Russian Academy of Sciences, 199178 Saint-Petersburg, Russia; konstantin.izrailov@mail.ru; 2 Department of Secure Communication Systems, The Bonch-Bruevich Saint-Petersburg State University of Telecommunications, 193232 Saint-Petersburg, Russia; 3 Department of Applied Mathematics and Information Technologies, Saint-Petersburg University of State Fire Service of EMERCOM of Russia, 196105 Saint-Petersburg, Russia; bmv1958@yandex.ru

**Keywords:** forensics, processor identification, code destruction, byte-frequency allocation, signature

## Abstract

Ensuring the security of modern cyberphysical devices is the most important task of the modern world. The reason for this is that such devices can cause not only informational, but also physical damage. One of the approaches to solving the problem is the static analysis of the machine code of the firmware of such devices. The situation becomes more complicated in the case of a Smart Home, since its devices can have different processor architectures (means instruction sets). In the case of cyberphysical devices of the Smart Home, the destruction of machine code due to physical influences is also possible. Therefore, the first step is to correctly identify the processor architecture. In the interests of this, a machine code model is proposed that has a formal notation and takes into account the possibility of code destruction. The article describes the full cycle of research (including experiment) in order to obtain this model. The model is based on byte-frequency machine code signatures. The experiment resulted in obtaining template signatures for the Top-16 processor architectures: Alpha, X32, Amd64, Arm64, Hppa64, I486, I686, Ia64, Mips, Mips64, Ppc, Ppc64, RiscV64, S390, S390x and Sparc64.

## 1. Introduction

Information security, the relevance of which in the modern world is unconditional, affects many areas that relate not only to countering relevant threats, but also to the study of their consequences [1,2,3]. So, after the accomplished fact of an attack on information resources, it is required to study how this attack was carried out, who was its initiator and what software and hardware was used. At the same time, it is necessary to take into account the fact that traces of a cybercrime can be deliberately hidden by an attacker, thereby making it difficult to find and punish him [4].

### 1.1. Relevance

Of particular criticality is the security of cyberphysical Smart Home devices, which actually produce physical influences under the influence of software in the human environment. Thus, violations of information security during the operation of these devices can have serious consequences for the life and health of people [5]. Moreover, the more cyberphysical devices are located in the area of a person’s presence, the greater the danger (in case of a successful attack) they will pose [6]. Preventing such consequences requires both counteraction to such attacks and their investigation, followed by preventive protection against them.

The investigation of cybercrimes is dealt with by the corresponding science–forensics [7]. Its main tasks are to construct (restore) the attack scenario and the chronology of events, collect traces of the intruder, compile his image (or corresponding more formalized models) and direct identification, propose preventive protection measures, and so forth.

One of the initial stages of forensics, obviously, consists of collecting information about the system, both the one at which the threat was directed (the victim) and the one from which the attack was carried out (the offender). Most attacks on information resources are carried out using executable files (typical examples of which are viruses, exploits, and logic bombs). The study of the machine code (hereinafter–MC) of executable files will allow one to understand the logic of their work, which, hypothetically, will make it possible to detect malicious code [8].

There is a sufficient number of methodological approaches and methods for the analysis of MC [9,10,11]. However, the primary task is to determine the architecture of the processor (hereinafter–Architecture), for execution on which this MC is intended [12,13,14]. This is necessary to select the appropriate tools designed to analyze only a specific set of machine instructions. In addition, knowledge about the Architecture will add information on the hardware component of the System (for example, some of them have processors of different Architectures; some can be used by an intruder for malicious actions). Architecture is understood not exactly the concept of the instruction set (RISC, CISC, etc.), but the used set of instructions (x86, PowerPC, MIPC, etc.).

Particular difficulty manifests itself in the analysis of MC of cyberphysical devices with various functional purposes. This follows from the fact that, depending on the purpose of the device, one can choose its own Architecture, adapted to the necessary tasks for the given requirements. For example, some cyberphysical devices require high computational efficiency (despite the resources spent), others demand low power consumption (at the expense of performance), and others claim high-quality computations (in the absence of time constraints).

Based on the designated aspects of the use of cyberphysical devices, including devices used for the functioning of Smart Homes, and the large heterogeneity of Architectures used in cyberphysical devices, as the current research area of this article, the study of the Architecture of CyberPhysical Devices of a Smart Home (CPDoSH) is highlighted [15]. There are the following reasons for this choice of area. First, the purpose of a Smart Home is to ensure human life, that is, most devices are continuously next to a human. Secondly, the devices perform qualitatively different tasks (they read the pulse on the hand, open the curtains, control the watering of the garden, play high-quality audio and video), that is, can be implemented using specialized Architectures.

To define the CPDoSH Architecture, it is required to develop an CPDoSH MC model (hereinafter, Model), which will allow creating algorithmic support for the corresponding identification method and its implementation. The model must be described in two forms:

- theoretical—having an analytical form, suitable for the entire set of CPDoSH MC;
- practical—allowing a demonstration of specific parameters (and characteristics) for the given CPDoSH Architectures.

Obviously, to obtain a practical form of the developed model, a number of experiments will be required.

### 1.2. Novelty

Thus, the main scientific result of the study, naturally possessing scientific novelty, is the analytical MC Model, adapted to identify its architecture. At the same time, the model allows one to analyze in a formal form the various operations of code destruction, that is relevant for the area of CDPoSH. This Model differs from the existing ones in the degree of detail and mathematical rigor of the description.

The article also poses and proves a hypothesis regarding the possibility of identifying an MC by its byte-frequency signatures. The novelty of the study is also confirmed by the fact that in the process of this proof, template Signatures for Top-16 Architectures are generated (according to the file sets from different assemblies of OS Gentoo).

This study is methodologically holistic for the following reasons:

First, the analysis of the subject area in terms of the CDPoSH MC metadata is carried out with a hypothesis about the identification of the Architecture. As a result of this analysis, a CDPoSH MC Model is formed.

Secondly, the work synthesizes a research software tool that allows one to check the validity of the hypothesis for identifying the Architecture.

Thirdly, a full-fledged experiment is carried out using both the resulting Model and the developed software. The results of the experiment make it possible to substantiate the hypothesis put forward.

### 1.3. Contribution

The theoretical significance of the work lies in building an MC model for a set of common architectures, taking into account the destructive actions characteristic of the area of CDPoSH. The problem of identifying the CDPoSH Architecture is also formalized in terms of the constructed Model.

The Model allows the use of formal tools for program analysis, putting forward hypotheses, building private (specialized for tasks) models of programs, and so forth. With the model, the process of code destruction is also characterized, and its basic typing is also given.

The practical significance of the work lies in the construction of specialized MC signatures for a set of common Architectures that are also used in CDPoSH. These signatures can be considered invariant from the purpose of the code, its size, and programming language.

A signature can be considered invariant with respect to more abstract characteristics of programs, such as development language, degree of optimization, functionality, and so forth. As a consequence, it is possible to collect a complete database of signatures for all existing types of processors.

The overall contribution of the study is aimed at developing tools for investigating cybercrime.

At the same time, without identifying the processor architecture (considered in this investigation), most methods of subsequent analysis will not be applicable.

### 1.4. Content

The structure of the article follows the sequence of research stages and is as follows.

Section 2 provides a review of articles related to the problem being solved—the creation of an analytical CPDoSH MC Model, adapted to identify its Architecture.

Section 3 describes the ontological model of the subject area of research, on the basis of which all subsequent research presented in this article is conducted. A diagram of the model and a description of all its elements are given.

Section 4 describes the progress of the study. At the first stage, the structure of the MC of programs is analyzed with the construction of an ideal Model. At the second stage, a CPDoSH MC Model is synthesized, taking into account possible destructive effects. At the third stage, a hypothesis is put forward regarding the possibility of identifying the CPDoSH Architecture by byte-frequency signatures. At the fourth stage, a research prototype for computing MC class signatures is created and described. At the fifth stage, with the help of this software, an experiment is carried out to obtain the Signatures of the CPDoSH MC classes. It is shown that the results of the experiment substantiate the hypothesis put forward.

Section 5 discusses the results obtained. A comparison of the proposed model with the closest analogs is made.

## 2. Analysis of Existing Review Works

We will review scientific works devoted to various methods aimed at classifying files, analyzing program code, detecting data areas with MC, and identifying its characteristics. All these results can be applied for the main investigation task—the analytical MC Model creation in the interests of the subsequent CPDoSH Architecture identification.

General approaches to the extraction of various metainformation from binary code have already been reflected in a number of scientific studies.

In [16], the Proof-of-concept of “genetic decompilation” is done. An approach is proposed that differs from the classical using the direction of transformation—from the original to the MC. So, instead of transforming the processor instructions to the constructions of the original programming language, the optimization of the variations of the source code is carried out by crossing and mutating them. Then, the source code is selected based on proximity to a given MC. Thus, the work is carried out inverse analysis of programs using genetic algorithms.

In [17], it is proposed to use byte histograms in conjunction with machine learning to identify areas with infected code in binary files.

In [18] the security of Samsung Smart Home applications is analyzed. Due to the placement of the execution environment (SmartThings) in the cloud, the source code of the individual applications (SmartApp) and device handlers is analyzed. The presence of flaws in the design of the products is indicated. Although this work is not directly related to MC modeling, it nevertheless substantiates the relevance of this problem. The development of this research is described in [19]. It also provides a solution for determining the byte order by the frequency of occurrence of individual byte values (0 × 0001 for Big Endian and 0 × 0100 for Little Endian). In particular, the WiiBin framework is used for this. The experiments carried out show the efficiency of the proposed approach.

In [20], a new ELF header format is proposed, which allows the specification of code for parallel execution on various threads of a multicore platform. Thus, each “.text” section will correspond to a piece of logically separated code. This problem arises due to the fact that initially the ELF and PE formats do not imply parallel code execution.

In [21], the problem of detecting errors in processor instructions during MC execution is solved (the most frequent of which is bit flipping, which changes the semantics of the instruction and often leads to a hardware failure). For this, it is proposed to use the Bloom filter in conjunction with the encoding/decoding scheme without executing an erroneous instruction.

In [22] it is proposed to identify the set of commands used in network devices (which do not have classic program headers) by applying SVM (Support Vector Machine). The dataset under study included many executable binaries for the Arm, Mips and PowerPC processors. The principle of feature selection is based on the representation of each 4-byte instruction as a tuple, according to which the subsequent training and classification takes place. A study similar to [18] is carried out in [23]. So, it is indicated that the addition of even one new CPDoSH increases information and physical risks for the entire human dwelling system.

In [24], the influence of the size of a register file on such indicators of the embedded software execution as its size, execution time and power consumption is estimated. An appropriate model has been developed, using which the compiler can select a sequence of instructions in accordance with the requirements for program execution. The results obtained can also be used to analyze applications in the design of ASIP (Application-Specific Integrated Processor). A similar algorithm for selecting user instructions for ASIP is developed in [25,26].

The Work [27] is devoted to the definition of steganographic software operating on the principles of the LSB (Least Significant Bit) Replacement Steganography algorithm. To identify such programs, the templates are used based on typical instructions used in the implementation of this algorithm.

In [28], an approach aimed at classifying programs into safe and malicious is presented. The fields of the header of the program in the PE format are used as features of each of the classes. Machine learning classifiers such as K-Nearest Neighbors, Gradient, Boosted Trees, Decision Tree, File large margin, Logistic Regression, Naive Bayes, and Random forest are compared. The advantage of the last classifier over the others is shown. A similar principle is described in [29].

Ref. [30] outlines a technology for detecting the PE-format malware based on text strings it contains—AMFP (Advanced Malware Forensics Procedure). Experiments have shown that AMFP can be used to identify operations in the program for working with the registry, network, files and other objects associated with unique text strings.

The solution given in [31] makes it possible to identify pirated software in the PE format. For identification, the following information is used, located in the fields of the PE-header: OptionalHeader.AddressOfEntryPoint, OptionalHeader.SizeOfInitializedData, OptionalHeader.SizeOfCode, OptionalHeader.DataDirectory [17]. If the values of the fields individually can be the same for different files, then their combination is considered unique for the malicious file. Similar approaches, but using machine learning, are described in [32,33,34].

In [35], a method is described for hiding malicious information at the end of PE headers, which does not affect the functionality of programs. In particular, it allows the creation of steganographic channels.

In [36], ELF programs are identified using the Minkowski metric. The frequency of occurrence of the most frequently used machine code instructions is used as the metric parameters. An internal audit of computer equipment is indicated as an area of application. The advantage of this technique is its high identification accuracy.

In [37], a method is described for identifying text files using the frequency of occurrence of words in the text files. The defined file types are aspx, bat, c, cpp, css, html, java, js, php, ps1, py, vb, and vbs. The following 7 machine learning algorithms are used for classification: K-Neighbors, Decision Tree, Random Forest Classifier, Support Vector Machine, Logistic Regression, Multinomial Naive Bayes and XGBoost.

In [38], the possibility of using hashes from data fragments to detect file remnants and compare them with malicious data contained in a database is investigated. Both block and sliding hashes are considered.

The work [39] is devoted to finding vulnerabilities in software using static analysis. ELF files are considered programs, and their templates are analyzed for vulnerability criteria. The search method is based on machine learning and vectorization of machine code instructions using Instruction2Vec (analogous to the well-known Word2Vec technology).

In [40], the most important problem of forensics is solved in the form of classifying parts of files. For this, it is proposed to use a hierarchical approach using a set of optimized SVMs. As features, 10 functions are selected that are calculated from the contents of the file (for example, the average byte value, Hamming weight, etc.).

In [41], to detect malicious software by its binary code, Simhash is used, a hash whose value is the same for close (but not necessarily identical) data. PCA (Principal component analysis) and linear transformation (simulating PCA using a neural network) are used to reduce the dimension.

In [42], it is proposed to use opcode frequencies to detect new viruses for which signature analysis is not suitable.

According to the review, only a small number of scientific articles are devoted to the issue of file classification, and even fewer—to the identification of the Architecture applicable to CPDoSH. Most of the existing work (partly included in the current review) is aimed at detecting malicious software, that is, solves a secondary problem when the Architecture is known and the necessary tool for working with it is selected. The works on the identification of the Architecture itself, although they describe promising ideas, nevertheless do not contain a comprehensive study on the subject of the final effectiveness. Thus, the scientific and technical problem stated in the current article is undoubtedly urgent. The problem does not have a full-fledged solution, and has not been studied from various aspects of practical application.

Based on the review, one can indicate the following prerequisites for solving the current problem. First, the analysis of files from CPDoSH firmware is quite efficient in ideal situations—for files without damage and with the correct header. Therefore, an attempt to disassemble “real” (different from ideal) programs for different processors will have a high level of errors (both I and II) and will require manual validation of the results. Secondly, the comparison of the hashes of the files under study (including the fuzzy ones, used to quickly assess the similarity of sets—SimHash, MinHash, FuzzyHash, etc.) with the reference database is efficient only if the latter is fully available. At the same time, due to the sufficient novelty of the Smart Home applications, such bases for CPDoSH are not yet sufficiently formed. Thirdly, a solution using byte-frequency signatures can be considered the most effective, although it may not be sufficiently resistant to the MC damage (which will require verification). This situation is especially relevant for CPDoSH, which are unlikely to have protection against vandalism or accidental damage, since they are consumer products, the price reduction of which is achieved in all possible ways. Fourthly, the applicability of machine learning methods in most works unconditionally speaks about the justification of this area and for solving the current problem. It would also be logical to assume that the artificial intelligence methods underlying the functioning of Smart Homes should also be aimed at ensuring the security of its cyberphysical devices.

Thus, it is interesting from a scientific and practical point of view to develop a solution for creating a Model suitable for the subsequent identification of the CPDoSH Architecture. Moreover, the Model must have the following characteristics:

- using the minimum number of parameters in the Model;
- full automation of work with the Model;
- simulation of MC, which can be protected from analysis and/or be partially destroyed;
- extensibility and adaptability of the Model for use in intelligent systems (for example, using machine learning methods).

## 3. Ontological Model of the Subject Area and Research Stages

Based on the general lack of research specifically in this subject area, it is necessary to first present the terminological basis for the identification problem of the CPDoSH Architecture, with which the current research will be described. In the interests of this, the following domain ontological model was developed (not related directly to the Model created in the current research).

**Figure 1:**
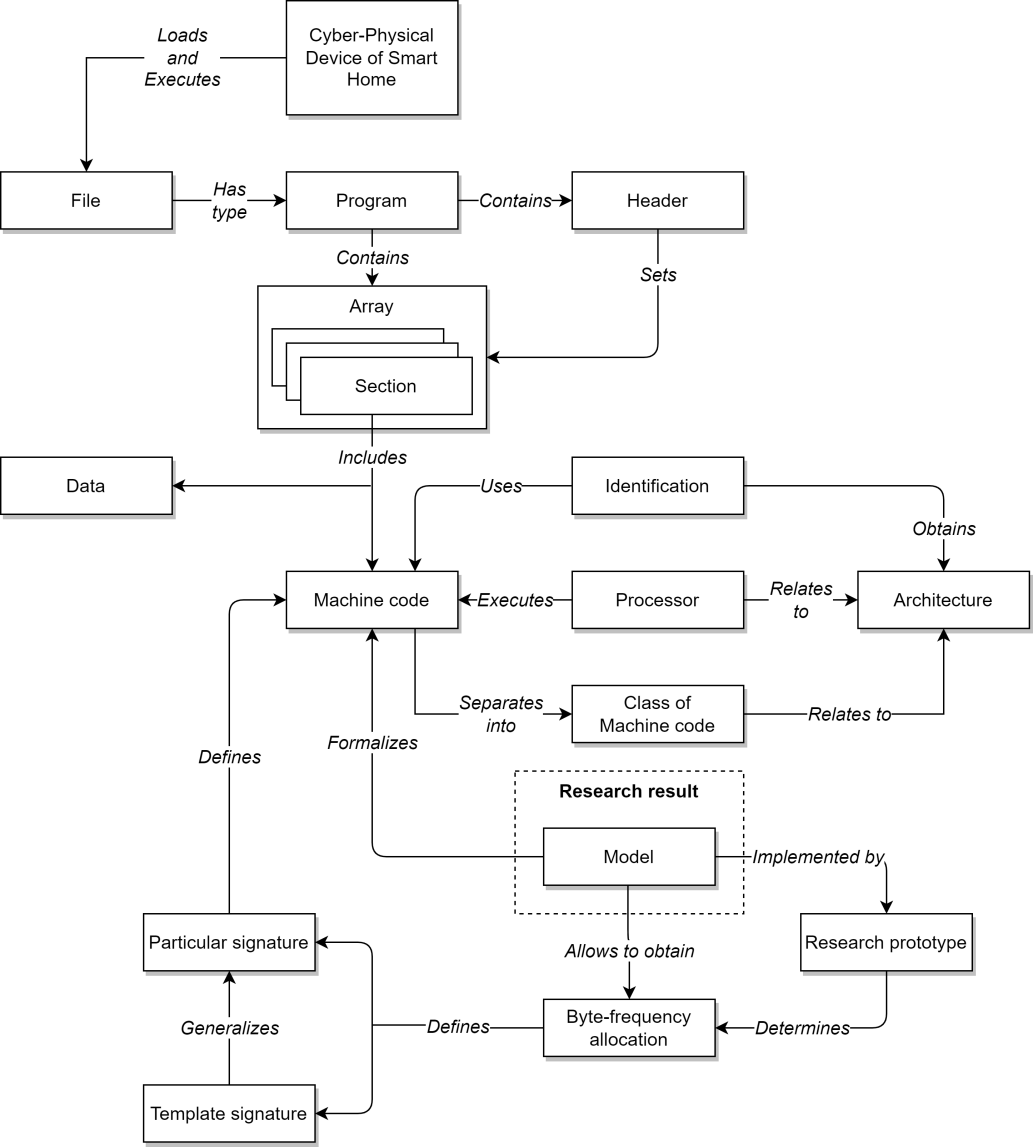
Ontological model of the domain.

The diagram of the ontological model of the subject area, which defines the basic concepts and their relationships, is shown in Figure 1.

Let us introduce the terms reflected in the domain model (see Figure 1) in the aspect of the problem being solved—the creation of the CPDoSH MC Model in the interests of identifying its Architecture:

- CyberPhysical Device of a Smart Home (or CPDoSH)—a device, which interaction with the physical world is determined by the program code (usually MC) running on its processor;
- File—information object of the CPDoSH storage; can be of various types that determine its purpose and data structure;
- Program—a type of the file that contains program code ready for execution in CPDoSH, as well as a header that defines meta information about the program;
- Header (programs)—an area with metadata at the beginning of the program, defining its structure (for example, sections with code and data) and launch parameters (for example, the type of Architecture); in the current context, two types are considered: ELF—for programs of the Windows OS family, PE—for programs of the Linux OS family (although others are possible);
- Section—an area with a lot of the same type of binary data contained in the program; in the current context may include MC and data for the operation of its algorithms;
- Machine code—program code (i.e., a formalized description of the logic of the algorithm) contained in the code section and ready for execution by the CPDoSH processor;
- Data—information contained in the data section and used by the CPDoSH processor (indirectly, through the program code) when executing the MC;
- Processor—an electronic unit (or integrated circuit) of CPDoSH that executes MC instructions; is an implementation according to some Architecture;
- Architecture (processor and machine code)—a formally given set of MC instructions executed by a family of CPDoSH processors;
- A class of machine code—a set into which an MC is divided, referring to one CPDoSH Architecture;
- Identification (Architectures)—the process of defining a specific type of CPDoSH Architecture; classically using a special field of the program header, and in this context using the MC program;
- Model (machine code)—a formalized description (both generalized for a group of programs and specific for a specific program) of MC, suitable for its identification; is the main scientific result of the research described in the article;
- Particular signature or just a signature (machine code)—the byte-frequency allocation of the MC of a single program for CPDoSH;
- Template signature (of Architecture and MC class)—byte-frequency allocation of MC of a certain class, collected from a fairly large number of programs of this CPDoSH Architecture; sufficiency in this case is determined by the fact that the Signature remains unchanged when new programs are added to the allocation; is a generalization of the frequent Signature;
- Byte-frequency allocation (of machine code)—allocation for binary recording of MC in the form of the number of bytes of each from 0 to 255 values, divided by the number of bytes of the maximum value; reflects the distinctive features of MC of a program of one Architecture from other programs and Architectures of CPDoSH; obtained from the Model and defines the Signatures of the Architectures (both particular and template);
- Research prototype (calculating the byte-frequency allocation)—a software tool prepared in the framework of the current research, which builds the byte-frequency allocation of a given program; allows one to calculate template signatures in this way.

This ontological model will allow one to avoid terminological confusion in the further consideration of the proposed approach and ongoing experiments, as well as to create a deeper understanding of this subject area.

Using the introduced terminological basis, we will describe the stages of the research described in the article.

Stage 1 analyzes the MC structure corresponding to programs running on the CPDoSH. The metadata highlighted in the MC allows constructing its “ideal” Model in an analytical form. The term ideal Model implies its applicability to the case when the MC was not subjected to both accidental and deliberate changes.

In terms of the Model, the following (target) research task is posed—the direct identification of the CPDoSH Architecture.

At Stage 2, the ideal Model is specialized for cases of MC destruction, which is especially important in the case of CPDoSH. To substantiate this, a real example can be given, when, as a result of an error in the heating subsystem of a Smart Home, there will be a critical temperature rise, which will lead to a fire [43]. As a result, some of the devices may be exposed to heat, thereby “spoiling” its CPDoSH program. Thus, when investigating the causes of an incident, the identification task of the Architecture will not be trivial (as, for example, in the case of the presence of the correct value of the corresponding field in the header of the PE or ELF format).

Then, a final analytical Model is proposed that reflects the CPDoSH MC even in the event of its destruction of any degree.

At Stage 3, the concept of an MC signature is analytically set, which allows one to give a more formalized record of the procedure for identifying the Architecture by the Model.

Then a hypothesis is put forward regarding the possibility of “distinguishing” Architectures using the MC Signature. The proof of the hypothesis substantiates the feasibility of the proposed of the CPDoSH MC Model (and subsequent identification of the Architecture).

At Stage 4, a research prototype for calculating the byte-frequency allocation of the CPDoSH MC (hereinafter—Prototype) is being developed. The Prototype architecture is beyond the scope of the current article solely dedicated to MC modeling. The prototype, on the other hand, is only an auxiliary tool.

In Stage 5, using the Prototype, a series of experiments is performed to calculate the byte-frequency allocation of the OS Gentoo assembly for Top-16 Architectures. Each of the Architectures can be applied to create the CPDoSH for various purposes, working conditions and required characteristics. For example, the embedded operating system eCOS [44] runs on a family of different hardware platforms, including the following: ARM, CalmRISC, FR-V, Hitachi H8, IA-32, Motorola 68000, Matsushita AM3x, MIPS, NEC V8xx, Nios II, PowerPC, SPARC, and SuperH.

As a result of experiments, the 16 byte-frequency signatures of the MC classes are practically formed. Each of the signatures statistically reflects a feature of a particular Architecture and can be used to directly identify them.

## 4. Research Progress

Let us describe further the progress of all 5 stages of the current research.

### 4.1. Stage 1. Machine Code Structure Analysis of Smart Devices

Let us analyze the structure of the MC of a program for CPDoSH and metadata (as information about the code itself), applicable to identify the Architecture. So, each program has a header of a certain structure with the information necessary for loading and execution. The most popular examples of headers are ELF (Executable and Linkable Format) and PE (Portable Executable); both define the way a program is displayed in memory, and are used in different operating systems (the first, as a rule, in Unix-like systems, and the second in Windows systems).

This header structure stores the following information, the most interesting from the point of view of MC identification. First, the structure contains directly the Architecture itself, on which the execution of the MC of the program is supposed. This field cannot be used in all 100% of cases—thereby completely solving the scientific and technical problem and achieving the research goal. The reason for this will be explained further. Secondly, sections with data are indicated in the structure, some of which are related to the executable code (sections are named as “.text”). The instructions of the processor, which architecture must be determined, are located in this section. Thirdly, modern programs can be 32-bit or 64-bit, which determines the bit depth of data and processor instructions for working with them. The corresponding field of the program header structure is intended for this. Fourthly, since most instructions consist of several bytes, their order in memory matters, which can go from high to low (denoted as big-endian and, as a rule, accepted by default) and vice versa—from junior to senior (denoted as little-endian and indicated on purpose). This is defined by another header field. It should be noted that, despite the seemingly obvious use of information about the Architecture from the header of the program for identifying the MC, this approach will not always work, and that is why the construction of a universal Model (according to the task of the current research) is relevant.

The inapplicability of using a header for identification is as follows.

Firstly, in a number of cases the Architecture in the header can be deliberately replaced with an incorrect one—precisely for the purpose of complicating its analysis during reverse engineering. This is justified by a large number of commercial CPDoSH developments, the uniqueness of the applied solutions, high competition in the field, which leads to the desire of developers to keep their know-how. At the same time, such a “substitution” will not affect the very performance of the program, since the MC will be executed on a specific CPDoSH, which has a known processor in advance.

Secondly, as a result of third-party factors (both accidental and malicious), the program can be destroyed (that is, some of its bytes will be changed or lost), which will simply lead to the “incorrectness” of this field of the header structure (and, possibly, the other fields).

Thirdly, the problem of identification is somewhat beyond the scope of a simple study of a full-fledged program, since for the CPDoSH it is an analogue of an embedded microprocessor system, which potentially does not even contain an Operating System. Consequently, for analysis, there can be an MC contained in another data set (even executed in memory) without a full-fledged program header. To analyze the same “raw” data in order to identify a malicious MC, you will first need to define its Architecture.

The result of the analysis of the structure of the program MC was the allocation of the following 4 types of metadata: Architecture, sections with MC, bit width and byte order. This metadata can be written in the form of Equation (1).

(1) $$\left\{\begin{array}{c} Model^{\prime }=<Architecture,Sections,DigitCapacity,Endianness>\\ Sections=\overset{N}{\underset{i=1}{⋃}}Section_{i}\\ Section_{i}=\overset{L_{i}}{\underset{j=1}{⋃}}Bytes_{i}\left[j\right]\\ DigitCapacity=\{32Bits,64Bits\}\\ Endianness=\{LittleEndian,BigEndian\} \end{array}\right.,$$

where $Model^{\prime }$—MC Model (the apostrophe means an ideal situation when the program header exists and fully corresponds to MC); Architecture—the CPDoSH Architecture indicated in the program header; Sections—a set of sections with MC; $Section_{i}$—a separate section; *N*—the number of sections; $L_{i}$—number of bytes in the i-th section; $Bytes_{i}\left[j\right]$—bytes of the *i*-th section (*j*—a index of bytes array); DigitCapacity—bit depth of processor data and instructions (can be 32-bit and 64-bit); Endianness—byte order (can be from junior to senior and from senior to junior).

Then the task of defining the Architecture (i.e., MC Class) becomes trivial, described using the Equation (1)

(2) $$Class=Identification^{\prime }:Model^{\prime }\to Architecture,$$

where Class—the required MC class; $Identification^{\prime }$—the MC identification procedure, extracting the Architecture from the header field of its $Model^{\prime }$ (the apostrophe also means getting the Class from the ideal Model).

The given Model fully reflects the MC, and the task of defining its Architecture is also written in an analytical form. However, neither the Model nor such identification can be fully applied for the CPDoSH, since (as emphasized earlier) the Smart Home device program differs from classical programs (for personal computers or server stations). Thus, MC in CPDoSH can be subjected to destructive changes that will not be reflected in the ideal Model (will not be taken into account).

The main actions of Stage 1 are presented using Algorithm 1 (in pseudocode form).
**Algorithm 1:** Pseudocode for Stage 1 algorithm
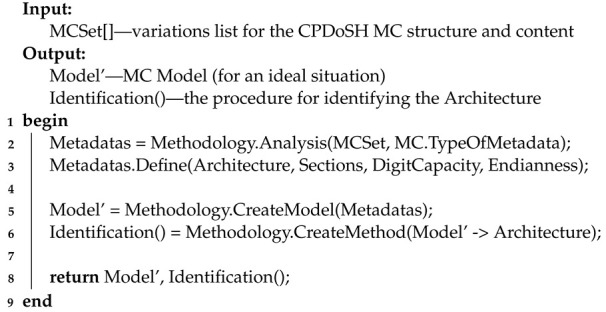


Algorithm 1 receives many variations of MC (MCSet) as input. The output algorithm returns the MC $Model^{\prime }$ and a hypothetical procedure for Identification its Architecture.

Line 1 starts the algorithm.

Line 2 examines the many options of MC from the point of view of the information they contain (MC.TypeOfMetadata). For this, the Analysis procedure is performed as part of the Methodology. As a result, MC metadata (Metadatas) is selected, which is applicable to define its Architecture.

Line 3 defines the selected Metadatas as Architecture, Sections, DigitCapacity, and Endianness.

In line 5, on the basis of the selected Metadatas, a $Model^{\prime }$ is built, which is a simplified MC rendering adapted to define the Architecture. To do this, the CreateModel procedure is performed as part of the Methodology.

In line 6, on the basis of the task (“convert (→) $Model^{\prime }$ of MC into its Architecture”), the Identification() procedure is determined. The procedure is intended, the operation of which should be based on the Model. To do this, the CreateMethod procedure is performed as part of the Methodology.

In line 8, the algorithm returns the generated $Model^{\prime }$ and the Architecture

Identification() procedure.

Line 9 ends the algorithm.

### 4.2. Stage 2. CPDoSH MC Model Synthesis

In the case of the destruction or absence of the program header (which is a special case of destruction), specialization (or refinement) of the Model will be required. Such a new Model of the CPDoSH MC (denoted without a apostrophe) can be written by applying to the old (ideal) with using of Equation (3).

(3) $$Model=Destruction(Model^{\prime }),$$

where Destruction—the procedure for destroying the program, which also leads to the destruction of the ideal $Model^{\prime }$ of its MC. Based on what was said before, we can distinguish 3 types of destruction procedure: deleting the correct value of the Architecture from the program header, deleting all structure fields except information about sections, and deleting the entire header in its entirety. These destructions can be analytically written using the Equation (4).

(4) $$\left\{\begin{array}{c} Destruction_{1}\left(Model^{\prime }\right):Architecture\to \emptyset \\ Destruction_{2}\left(Model^{\prime }\right):Architecture,DigitCapacity,Endianness\to \emptyset \\ Destruction_{3}\left(Model^{\prime }\right):Architecture,DigitCapacity,Endianness,Sections\to \emptyset \end{array}\right.,$$

where $Destruction_{k}$—type *k* destruction, *∅*—the designation for converting the header field to an incorrect value.

So, the first type of destruction corresponds to the situation of deliberate change of the CPDoSH Architecture in the program header to the wrong one. The second type means the absence of a header with a clearly defined range of MC instructions (since the sections are saved). The third type is identical to a set of some data potentially containing MC, the identification of which must be carried out. If in the case of the destruction of the first and second types, the information about the sections remains, and, therefore, the bytes of MC for identification can be used, then in the case of the third type it will not even be possible to select the MC, since it will “mix” with the data other sections (for example, containing string constants). However, there are techniques for detecting MC in a dataset, which will allow solving this problem of allocating bytes of MC [45].

Based on this, despite the type of destruction of the program, the final Model of the MC can be written in the form of a set of bytes of its instructions (see Equation (5)).

(5) $$\left\{\begin{array}{c} Model=\overset{N}{\underset{i=1}{⋃}}\overset{L_{i}}{\underset{j=1}{⋃}}Bytes_{i}\left[j\right] \end{array}\right.,$$

which is metadata, invariant in some sense to destruction. Thus, the identification task is reduced to extracting new meta-information from the given Model, which can be compared with the Class of the CPDoSH MC. Since the procedure and the result of such identification should not depend on the MC size (since the Class is determined only by the processor for MC), it is necessary to “get rid” of the length parameters in the formula—*N* and $L_{i}$. The latter can be provided by using for identification the byte-frequency allocation of its instructions—the frequency of “meeting” bytes of instructions in all the executable code contained in the file.

The main actions of Stage 2 are presented using Algorithm 2.
**Algorithm 2:** Pseudocode for Stage 2 algorithm
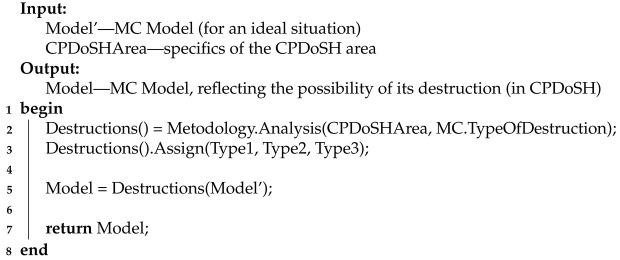


Algorithm 2 receives a Model for an ideal situation ($Model^{\prime }$) and CPDoSHArea as input. The output algorithm returns a Model for a situation more typical for CPDoSH, when MC destruction is possible.

Line 1 starts the algorithm.

Line 2 examines the specifics of CPDoSHArea from the standpoint of possible ways of destructive effects on the MC (MC.TypeOfDestruction). For this, the Analysis procedure is performed as part of the Methodology. As a result, the procedures Destructions() are distinguished, which are applicable for the destruction of the MC.

In line 3, the selected MC destruction procedures are divided into 3 types—Type1, Type2 and Type3.

In line 5, the selected MC Destruction() procedures are applied to $Model^{\prime }$. This creates the Model applicable to the CPDoSH area.

In line 7, the algorithm returns the generated Model.

Line 8 ends the algorithm.

### 4.3. Stage 3. Assumption the Identification Hypothesis of the CPDoSH Architecture

The byte-frequency allocation of MC instructions by analogy with the well-known works [46] will be called the byte-frequency signature of MC (i.e., the previously mentioned Signature, see Figure 1. Thus, the Signature will display the MC features that do not depend on the method of its structural organization—the location of instruction bytes, their length, the logic of the algorithms being implemented, constant data, and so forth.

Formally, the signature of any file can be written as an array of frequencies of values of its bytes from $2^{8}=256$ elements using Equation (6).

(6) Signature=Frequency[256](Bytes),

where Bytes—a set of bytes (in no particular order), and the frequency Frequency[i]—is normalized to the maximum value, that is, it is defined as the number of bytes with the value *i* in relation to the maximum number of bytes Count[i] (see Equation (7)).

(7) $$Frequency\left[i\right]=\frac{Count[i]}{max(Count[0],...,Count[255]}.$$

The procedure for determining the Class using the identification procedure can be written using Equation (8).

(8) $$\left\{\begin{array}{c} Class=IdentificationOne:Model\to Signature(RawBytes)\\ RawBytes=\overset{N}{\underset{i=1}{⋃}}\overset{L_{i}}{\underset{j=1}{⋃}}Bytes_{i}\left[j\right] \end{array}\right.,$$

where IdentificationOne—the procedure for the precise determination of the CPDoSH MC Class, which extracts (→) from its Signature the Model, using the set of all bytes of MC (RawBytes); *N*—number of sections of the program with MC; $L_{i}$—number of bytes in the *i*-th section; $Bytes_{i}\left[j\right]$—the *j*-th byte in the *i*-th section.

Proceeding from the set goal and the formalized MC Model, the research task is reduced to the creation of a method for comparing the Signature of a sample of the CPDoSH MC and its Class. In the interests of this, we put forward the following hypothesis (hereinafter—Hypothesis 1):

**Hypothesis** **1.** *Many machine code files running on a single processor architecture have their own unique frequency signature that is different from the rest.*

Hereinafter, for simplicity and in the interests of the future experiment, we will assume that each copy of MC (possibly consisting of many sections) is stored in the CPDoSH in a separate file.

This hypothesis can be formally written using Equation (9).

(9) $$\left\{\begin{array}{c} Signature^{Class_{i}}=BuildSignature\left(\overset{F}{\underset{f=1}{⋃}}Model_{f}^{Class_{i}}\right)\\ \forall i,j\left(i\neq j\right),Signature^{Class_{i}}\neg \approx Signature^{Class_{j}} \end{array}\right.,$$

where $Signature^{Class_{i}}$—Signature of the Models set of CPDoSH MC for one $Class_{i}$; BuildSignature()—procedure for constructing a Signature of CPDoSH MC from a set of $Model_{f}$ of the same Class; *F*—the number of Models of this Class; moreover, the Signature of one $Class_{i}$ is significantly different (¬≈) from the Signature of another $Class_{j}$. The success of the confirmation of the Hypothesis will speak of the capability of identifying the CPDoSH Architecture.

The main actions of Stage 3 are presented using Algorithm 3.
**Algorithm 3:** Pseudocode for Stage 3 algorithm
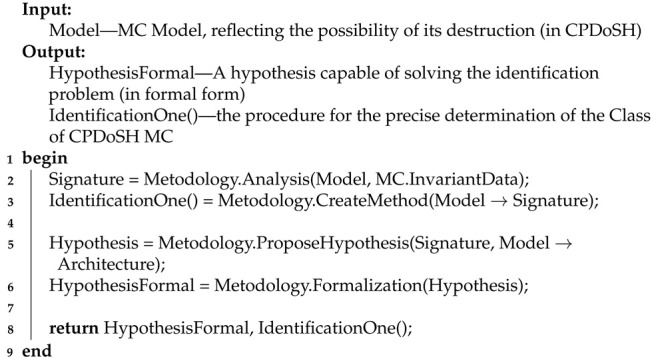


Algorithm 3 receives a Model for a situation with the possibility of destruction of MC as an input. The output algorithm returns a Hypothesis that can help in solving the problem of identifying the Architecture, and procedure IdentificationOne() for precise determination of the Class of CPDoSH MC.

Line 1 starts the algorithm.

In line 2, the Model is considered from the position of selecting the data that are resistant or invariant to destruction of MC (MC.InvariantData). For this, the Analysis procedure is performed as part of the Methodology. As a result, the concept of Signature is introduced, which reflects the MC features that do not depend on the method of its structural organization and that minimizes dependence on destructive influences.

In line 3, on the basis of the task (“convert (→) Model of MC into its Signature”), the operation of which is based on the selected invariant data, the IdentificationOne() procedure is determined. The procedure, like Identification(), is intended. To do this, the CreateMethod procedure is performed as part of the Methodology.

Line 5 puts forward a Hypothesis based on the introduced concept of Signature. The purpose of the Hypothesis is to solve the problem of separating the Architecture from the Model of MC. Hypothesis is in text form. For this, the ProposeHypothesis procedure is performed as part of the Methodology.

Line 6 converts the Hypothesis into a more formal form (HypothesisFormal). For this, the Formalization procedure is performed as part of the Methodology.

In line 8, the algorithm returns the advanced and formalized HypothesisFormal (the main of which is Signature), as well as the procedure for determining the Class of the CPDoSH MC. The algorithm also returns a IdentificationOne() procedure.

Line 9 ends the algorithm.

### 4.4. Stage 4. Creation of a Prototype for Constructing CPDoSH MC Signatures

To verify the Hypothesis, it is necessary to construct the averaged byte-frequency allocations of MC of all classes, that is, the Class Signatures of the CPDoSH MC. In the interests of this, a research prototype was developed, adapted to work with the CPDoSH MC. Although the description of the prototype and its basic testing is beyond the scope of the current work on modeling of MC, we will give brief information about the Prototype.

The prototype is a console software tool that runs in the OS Windows environment.

For the development of the Prototype, the Microsoft Visual Studio environment (version 2019) was chosen, which is one of the undisputed leaders in the field of IT engineering. Including for this reason, the development language was C# (obviously supported in the environment).

Despite the high and constantly growing popularity of the Python language, especially when developing programs using machine learning, it was not applied for a number of the following subjectively negative signs: focus on ease of writing code, which for programmers with more experience rather complicates development, because it requires memorizing new (and not always logical) “simple” constructions; “Related proximity” of C# and Microsoft Visual Studio, which is naturally reflected in the use of the former in the latter (support for refactoring, ease of debugging, etc.); negative author’s experience of using scripting languages (Python, Ruby, etc.) versus those built on “pure” bytecode (C#, Java), as well as the results of independent studies that show higher speed and less resource consumption of the second group of languages compared to the first, which also influenced the final choice.

The main purpose of the prototype is to build byte-frequency signatures based on OS Gentoo assemblies for a set of Architectures.

The prototype works in the following 3 modes:

- Mode 1 (preparatory): unpack the OS Gentoo build image into a separate directory for further processing);
- Mode 2 (optimization): deleting from unpacked (after Mode 1) files that are not involved in the construction of signatures, since they are not programs;
- Mode 3 (main): processing of MC in unpacked (after Mode 1) and optimized (after Mode 2) files contained in programs, and construction of template Signatures for the CPDoSH MC by their bytes.

The Prototype is a console application with the following startup argument format (MCArchIdent.exe is the name of the application executable):

- for Mode 1:
  
> MCArchIdent.exe UnpackMode ArchiveFile UnpackDir
  where UnpackMode is an indication of the current operating mode, ArchiveFile is the path to the archive file for unpacking, UnpackDir is the path to the directory for unpacking the archive;
- for Mode 2:
  
> RemoveMode SomeDir
  where RemoveMode is an indication of the current operating mode, SomeDir is the path to the directory for removing unnecessary files (except for ELF and PE programs);
- for Mode 3:
  
> MCArchIdent.exe SignatureMode SomeDir SignatureFile ElfArId PeArId
  where SignatureMode is an indication of the current operation mode, SomeDir is the path to the directory with programs for building a template Signature; SignatureFile—path to the file for saving the template Signature; ElfArId—a numeric identifier of the Architecture to check in the ELF program header; PeArId—a numeric identifier of the Architecture to be checked in the PE header of programs.

Here is a brief report on the implementation of all three modes of operation. For Mode 1, Prototype was executed with the following arguments:


> MCArchIdent.exe UnpackMode D:/stage3-alpha-20200215T160133Z.tar.bz2
  X:/Alpha/


As a result of execution, the following log will be displayed (hereinafter, for simplicity, only a part of it is shown, the rest of the lines are replaced by symbols “…”):


[Unpack Archive] stage3-alpha-20200215T160133Z.tar.bz2:/ -> X:/Alpha/
Unpack Bzip: ’D:/stage3-alpha-20200215T160133Z.tar.bz2’ -> X:/Alpha/
Unpack Tar: ’X:/Alpha/stage3-alpha-20200215T160133Z.tar’ ->
  X:/Alpha/!__stage3-alpha-20200215T160133Z.tar/
Unpack Tar: ’X:/Alpha/!__stage3-alpha-20200215T160133Z.tar/usr/lib/
  python2.7/test/testtar.tar’ ->
X:/Alpha/!__stage3-alpha-20200215T160133Z.tar/usr/lib/python2.7/test/
  !__testtar.tar/
Unpack Tar: ’X:/Alpha/!__stage3-alpha-20200215T160133Z.tar/usr/lib/
  python2.7/test/PaxHeaders.138508/testtar.tar’ ->
  X:/Alpha/!__stage3-alpha-20200215T160133Z.tar/usr/lib/
  python2.7/test/PaxHeaders.138508/!__testtar.tar/
Unpack Tar: ’X:/Alpha/!__stage3-alpha-20200215T160133Z.tar/usr/lib/
  python3.6/test/testtar.tar’ ->
X:/Alpha/!__stage3-alpha-20200215T160133Z.tar/usr/lib/python3.6/test/!
  __testtar.tar/
…


Thus, the OS Gentoo Alpha image will be unpacked into a separate directory.

For Mode 2, Prototype was executed with the following arguments:


> MCArchIdent.exe RemoveMode X:/Alpha/


As a result of execution, the following log will be displayed:


[Remove extra files] X:/Alpha/


This will remove all non-ELF and non-PE files from the unpacked OS Gentoo Alpha image.

For Mode 3, Prototype was executed with the following arguments:

		
[Collect signature] D:/Alpha/ -> Alpha.sig


Thus, for MC programs in ELF format with the Architecture identifier 0x9026 (i.e., for Alpha) a template signature will be built from the X:/Alpha/ directory, which will then be saved in the Alpha.sig file.

The above testing shows the basic performance of the Prototype.

The main actions of Stage 4 are presented using Algorithm 4.
**Algorithm 4:** Pseudocode for Stage 4 algorithm
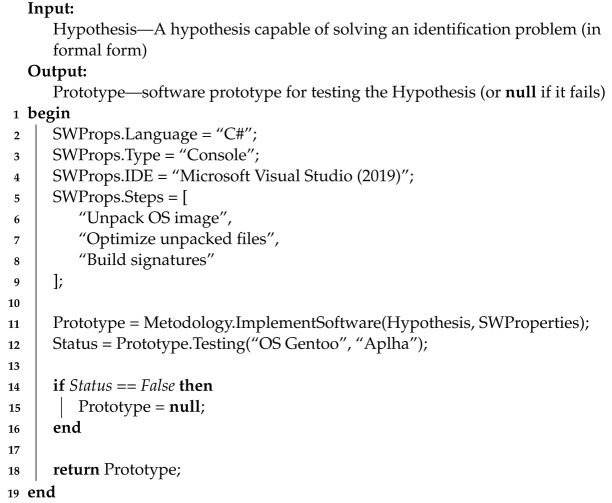


Algorithm 4 at the input receives the previously advanced Hypothesis regarding the use of MC signatures to identify its Architecture. The output algorithm returns the Prototype used to test the Hypothesis. In the case of the basic inoperability of the Prototype (i.e., based on the results of its testing), the algorithm will return the entity of the absence of an object—null.

Line 1 starts the algorithm.

From lines 2 to 9, using the fields of the SWProperties construction, the parameters of the software tool are determined according to which the prototype must be created.

Line 2 specifies the development Language of the Prototype—C#.

Line 3 specifies the Prototype Type—a console application.

Line 4 specifies the integrated development environment (IDE) for the Prototype—Microsoft Visual Studio 2019 release.

Line from 5 to 9 indicates the main steps of the Prototype (written in pseudocode as a text list of subtasks).

Line 11 creates a Prototype that validates the Hypothesis. Development requirements are specified in the SWProperties construct. To do this, the CreateMethod procedure is performed as part of the Methodology.

Line 12 puts forward the basic Testing of the OSGentoo Build Prototype for the Alpha Architecture. The test result is entered into the Status variable.

Line 14 checks the results of testing the Prototype (in Status variable).

Line 15 starts the execution branch for the Prototype test failure case. As a result, the Prototype object is deleted (equated to Null).

Line 16 ends the conditional execution started in line 7.

On line 18, the algorithm returns the created Prototype (or null if it fails).

Line 19 ends the algorithm.

### 4.5. Stage 5. Conducting an Experiment to Verify the Hypothesis

To conduct an experiment to test the Hypothesis, as sets of files from MC, we used OS Gentoo assemblies, Stage 3 assemblies for the following set of Architectures (close to the naming on the official OS website [47]): Alpha, X32, Amd64, Arm64, Hppa, I486, I686, Ia64, Mips, Mips64, Ppc, Ppc64, RiscV64, S390, S390x, Sparc64. All of these Architectures can be used in CPDoSH work. Postfixes 32 and 64 in the names of Architectures denote the bitness, and if it is absent, the names of the Architectures are well-established and/or trademarked (for example, S390 corresponds to the 32-bit version of the processor, and S390x—to the 64-bit version).

It should be noted that on the official OS Gentoo site there were assemblies of other variations of these Architectures (for example, 32-bit Spark, the previous version of Hppa). However, as a preliminary study has shown, such variations have a sufficiently high proximity of byte encoding of instructions, which does not allow using their Signatures for accurate identification. Therefore, they were excluded from further consideration. Such variations include assemblies for Arm with different byte order and hardware support for floating point or Mips with 32 and 64 bit for different byte order, as well as HPPA for different bit order. However, this is not a disadvantage, since the Signature of any variation of this Architecture is significantly different from the Signatures of other Architectures. With equal variations of assemblies of two bits with the proximity of their Signatures, it was 64-bit that was taken, as reflecting the modern trend in the development of information technologies to increase the size of the processed data. In the case of variations for different byte ordering, the most popular was taken—*big endian*; however, as noted, the byte order should not affect the Signature, since the byte order remains the same.

A brief transcript of the given Top-16 Architectures (including their variations, which are considered a separate Architecture in this research), is as follows:

1. Alpha—for Alpha (only 64-bit);
2. X32—for 64-bit AMD when working with 32-bit numbers;
3. Amd64—for 64-bit AMD;
4. Arm64—for 64-bit ARM;
5. Hppa64—for 64-bit Hewlett Packard (with Precision Architecture);
6. I486—for 32-bit Intel up to Pentium Pro and II;
7. I686—for 32-bit Intel for Pentium Pro, II, etc.;
8. Ia64—for 64-bit Intel Itanium;
9. Mips—for 32-bit MIPS;
10. Mips64—for 64-bit MIPS;
11. Ppc—for 32-bit PowerPC;
12. Ppc64—for 64-bit PowerPC;
13. RiscV64—for 64-bit RISC-V;
14. S390—for 32-bit IBM System;
15. S390x—for 64-bit IBM System;
16. Sparc64—for 64-bit SPARC.

Each of the list of Architectures corresponds to the Class, which must be identified by the MC CPDoSH.

All OS assemblies, except for Mips, had the openrc label, as well as the build time—2020–2021, and, therefore, were close in functionality. The OS Gentoo for the Mips Architecture was taken from a repository with experimental assemblies, which is quite acceptable, since the main task of using assemblies was to obtain a set of complete programs with a large number of MCs for a certain processor—to create a statistically correct Signature.

Each assembly looked like a compressed TAR container (containing other nested containers) and sizes from 157 MB (for Sparc64) to 324 MB (for Alpha). Such a spread in the sizes of assemblies (with the proximity of the functionality implemented in them) can be explained by the different number and size of files included in the composition that are not related to programs with MC. The overwhelming majority of programs within assemblies were in ELF format.

In addition to the significant size of the sample (even taking into account the fact that not all files in assemblies contain MC), it can be considered quite diverse in terms of all possible variants of Signatures, since Gentoo is an OS and, therefore, contains the implementation of a fairly wide set of functionality. The extraction of files from each assembly was carried out by another module, also included in the Prototype.

The name of the OS Gentoo assembly file and their sizes, as well as the sizes of the MC obtained from the sections of all the extracted executable files for each of the Top-16 Architectures, made up the values given in Table 1. This dataset can be obtained from the Internet resource by reference [48]. For convenience, the table also contains numeric identifiers of the Architectures, which will be used below.

Following Table 1, on average, one byte value accounts for approximately $\frac{157+324}{2}\approx 240$ MB of data (ranging from ≈157 MB for Sparc64 to ≈324 MB for Alpha), which can be considered sufficient to identify a general pattern in the byte-frequency allocation of MC Classes.

Using the Prototype sequentially in 3 modes for each of the OS Gentoo assemblies will allow obtaining Signatures of 16 MC classes, suitable for identifying of CPDoSH. Such a set of Signatures represents a Model of the CPDoSH in a practical form, specialized for one Architecture from the Top-16.

**Figure 2:**
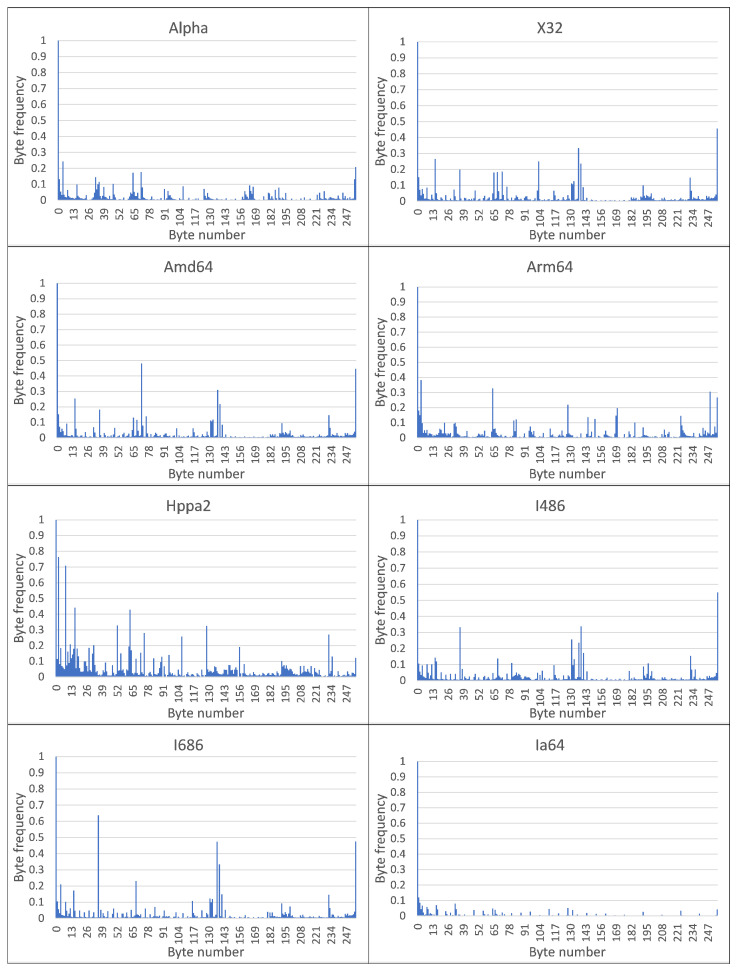
Byte-frequency signatures of MC classes for Top-16 CPDoSH Architectures (for Alpha, X32, Amd64, Arm64, Hppa64, I486, I686 and Ia64).

**Figure 3:**
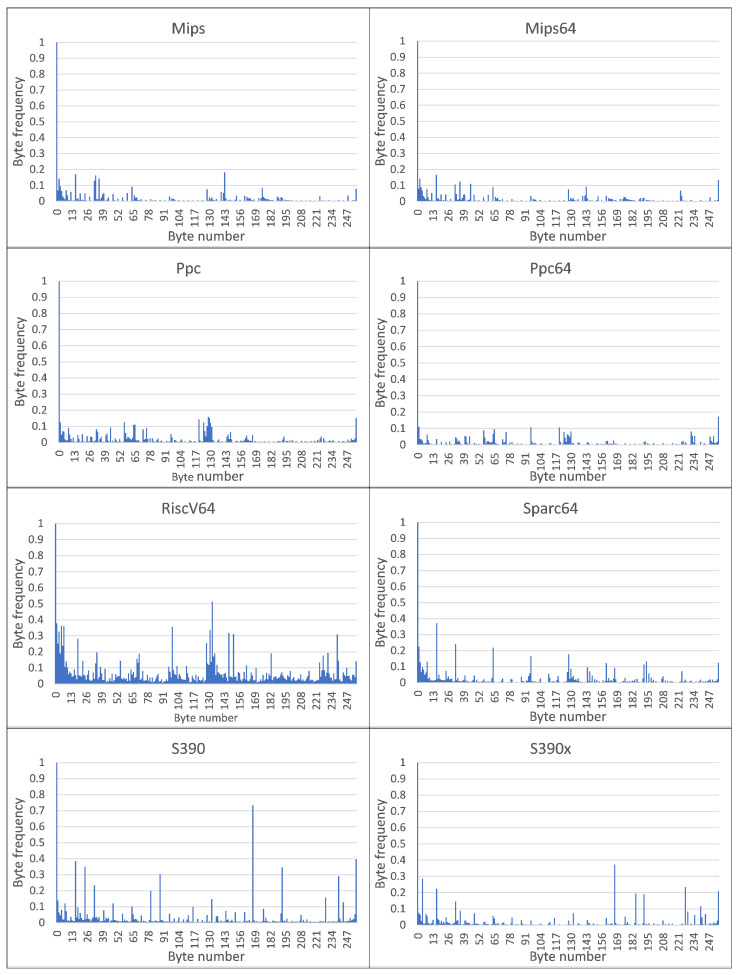
Byte-frequency signatures of MC classes for Top-16 CPDoSH Architectures (for Mips, Mips64, Ppc, Ppc64, RiscV64, S390, S390x and Sparc64).

The resulting MC CPDoSH Class Signatures are shown in Figure 2 and Figure 3.

A visual analysis of the graphical representation of histograms for the Class Signatures of the CPDoSH MC (see Figure 2 and Figure 3) allows us to draw the following conclusions.

Firstly, the Signature of each Class has a clearly distinguished peak of the maximum height at a byte value of 0, which is explained by the use of this value as the standard in many cases—for example, during initial memory initialization.

Secondly, each Class is characterized by a high frequency of occurrence of a byte with a value of 255. It is apparently due to its frequent use for service needs; for example, a value of −1 is corresponding to the unsigned form of writing the number 255.

Third, the frequency for the value 0 is about 2–5 times higher than the frequency for the value 255, which can be explained by the general ratio of the use of these numbers in the program code.

Fourthly, 32-bit and 64-bit Architectures that are quite similar in their principles of functioning, differing only in the size of the data being operated (and, accordingly, in the extended set of commands), have different Signatures, which should allow identification for them as well (for example, Mips vs Mips64, S390 vs S390x, etc.). However, as it was noticed, similar variations of Architectures, but with indistinguishable Signatures, were excluded from the Top-16 in advance.

Fifth, visual analysis of histograms allows one to assert about the general difference in frequency peaks (and not only in their amplitude for some values), which should increase the identification accuracy.

Sixth, RiscV64 has a rather interesting signature “portrait”, which is characterized by a larger number of peaks, noticeable in magnitude, from 0.0 to 0.2 (this is clearly seen from the large volume of the painted surface). This can be explained by the specification of the Architecture instructions, which allows uniform use of bytes for encoding for typical programs. Thus, this distinctive feature will play into the hands of the identification of this Class.

Seventh, the most weakly expressed Signature is possessed by the Architecture for Ia64, since all of its peaks, except for the 0-th one, do not exceed 0.1 (i.e., 10% of the maximum); it is the success of the identification of this Architecture that will be one of the criteria for the success of the identification.

Summing up the preliminary results, we can make a general conclusion that the Signature of MC for a some Architecture differs significantly from the Signature of MC for other CPDoSH Architectures. Consequently, the hypothesis put forward can be considered confirmed.

The exclusion of Architectures with similar Signatures is not critical, since they can be combined into one, since they, in fact, represent the same set of processor instructions. At least in the vast majority of cases, static analysis methods and tools will work exactly the same for such variations.

Thus, the Hypothesis can be considered completely correct and, therefore, the use of Signatures for assigning of the CPDoSH MC to the appropriate Class is legitimate, which confirms their application for identification.

The main actions of Stage 1 are presented using Algorithm 5.
**Algorithm 5:** Pseudocode for Stage 5 algorithm
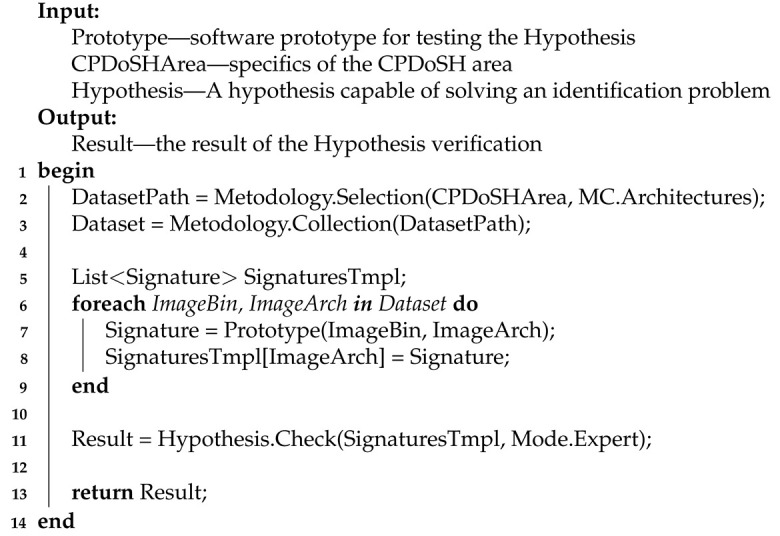


Algorithm 5 receives a Prototype for checking the Hypothesis, the specifics of the CPDoSHArea and the Hypothesis about the identification of the Architecture itself. The output algorithm returns the Result of the Hypothesis verification.

Line 1 starts the algorithm.

Line 2 discusses the specifics of CPDoSHArea for the selection of OS images for different Architectures (MC.Architectures). For this, the Selection procedure is performed as part of Methodology. The paths to the OS images are stored in the DatasetPath object.

In line 3, a Dataset is formed from the selected OS images by DatasetPath object. For this, the Collection procedure is performed as part of Methodology.

Line 5 defines a List of template Signatures—SignaturesTmpl.

Line 6 begins a loop to iterate over the OS images (ImageBin) and their Architecture (ImageArch) in the Dataset.

Line 7 calculates the template Signature for the current OS image and its Architecture.

In line 8, the computed signature is added into the list of template signatures.

Line 9 ends the loop started on line 6.

Line 11 performs Hypothesis verification for the list of template signatures calculated using the Prototype. The essence of the Check is to compare these signatures and search for distinctive features in them. The comparison is carried out in an expert way (Mode.Expert). The presence of such features will make it possible to reliably refer both the signature itself (and the set of MCs corresponding to it) to different Architectures. The result of the Hypothesis verification is entered into the Result variable.

On line 13, the algorithm returns the Result of the Hypothesis verification.

Line 14 ends the algorithm.

## 5. Discussion

To analyze the functionality of the proposed MC model (applicable, among other things, for CPDoSH), let us compare it with the closest analogs.

In [49], an approach is proposed for constructing heuristic malware detectors. The approach focuses on handling static position-dependent functions that take into account the specifics of the object file format. It is shown that the position-dependent features of the application, extracted at the stage of static analysis of executable files, can be used quite effectively to detect malicious programs.

In [50], a model for identifying meta-information about the compiler by the binary code of the program is described. The model is based on neural modeling. The experiments show the high-precision recover of the following meta-information: compiler family, optimization level, compiler version.

In [51], a model based on a deep neural network is proposed to improve the binary analysis of the binary code of Android programs. The problem of identifying the boundaries of function addresses is solved. Experiments have shown satisfactory performance values: precision, recall and F-measure are approximately equal to 0.75–0.80.

In [52] the problem of identification of polymorphic variants of viruses is solved. It is pointed out that the previous methods used a flow graph and a signature tree. In the proposed approach, it is suggested to use a hierarchical hidden Markov model that increases the identification accuracy. The model reflects the self-similarity and hierarchy of signature families. Experiments have shown a low number of Type I and Type II errors.

In [53], the problem of detecting vulnerabilities in program code is solved. The search for vulnerabilities is carried out using a special embedding model. A feature of the solution is its application for various processor architectures.

In [13], a method for searching for malware using deep learning is proposed. The binary code of the programs is used for both training and testing. The problem of retraining such models of neural networks is pointed out. To counteract this, an additional ensemble of algorithms is applied.

The work [54], similar to the current research, is devoted to the protection of IoT devices from malware. For this, static analysis of the binary code is used. The multi-architecture of solutions for IoT is emphasized. The paper proposes a method for detecting malicious programs based on a graph neural network. Each binary program is represented as an architecture-independent function call graph. Experiments are being carried out on the programs for 5 Architectures.

In [55], the problem of detecting the similarity of the binary code of programs is solved in the interests of information security. As in [53], the diversity of IoT device architectures is emphasized, which does not allow the use of classical comparison methods. In the interests of this, it is proposed to use the Abstract Syntax Tree, which has a large content of semantic information, weakly depending on the Architectures. The proposed solution (Asteria) is built on the basis of deep learning.

Based on the correct research task (identification of the MC CPDoSH Architecture), we introduce the following criteria for comparison with analogues based on the available functionality (including the rationale for this choice):

- A—Applicability to several Architectures, since CPDoSH can run on a whole set of “non-classic processors” (i.e., except for Intel and AMD);
- B—Possibility of using in methods based on artificial intelligence, since the huge volumes of MC for analysis, the high labor intensity of manual analysis, the lack of the required number of code security specialists, errors in the operation of automatic tools (based on expert rules) require the use of new intelligent technologies;
- C—Use of unique signatures (i.e., pre-assembled and verified template databases), since the definition of Architectures from a known set (for example, in a database) is required, which can be updated; each Architecture from the set must have a form that is different from the others, which can be interpreted as its unique signature;
- D—Restoration of meta-information about MC, since the main purpose of the Model is to identify the Architecture of MC, which is itself the required meta-information, and can also be “collected” from auxiliary meta-information;
- E—Applicability in the tasks of static analysis of MC that go beyond the search for vulnerabilities, since the information security of the CPDoSH is carried out not only by detecting malicious code; for example, it is necessary to check the compatibility of instructions in the MC image with the hardware of the device being used, to take into account the influence of the physical features of the used Architecture (for the MC being executed) on the functioning of the CPDoSH as a whole, to track own vulnerabilities of the Architectures implementation, and so forth.;
- F—Operability after the destruction of MC, since a feature of cyber-physical devices (including the CPDoSH) is their susceptibility to external physical influences, which also lead to information destruction of MC.

The results are presented in Table 2.

The analysis of the comparison results allows us to assert the advantage of the proposed Model (6 points) over analogues (1.5, 2.5, 3, 3.5, 4 and 4.5 points). At the same time, the gap from the closest analogue [49] is 1.5 (i.e., 25%) points, which can be considered a significant superiority.

At first glance, it may seem that the analytical Model is obtained in a too complex way, since it could be assumed empirically. However, such “guesses”, obtained on the basis of expert opinion, rather than considerations of strict logic, often lead to false knowledge. The proposed method for creating a Model is based on a more rigorous methodology than similar ones. This is also the difference between the conducted research and the existing ones—the correctness of creating a model through confirmation of the Hypothesis.

In most cases, the Architecture in the program is already indicated in the file header. However, in addition to the possible destruction of this meta-information, the field with the Architecture can be deliberately changed by the developer himself, as part of protecting the data from reverse engineering. This fact, which at first glance does not inspire confidence, is confirmed by the experience of researching binary firmware of telecommunication equipment from well-known manufacturers.

Often, manual research is sufficient to define an Architecture, for example, by discovery and analysis in products such as IDA Pro. Thus, enumeration of various sets of processor instructions and expert examination of the disassembled code will allow choosing the correct Architecture. However, this approach will not work for areas of binary data, in which there is no markup not only about the sections with the code, but also about the number of Architectures used. So, instructions for one Architecture can be mixed in the image, then processor-independent data, then instructions for another Architecture, then just a random set of data, and so forth. This situation is quite typical for monolithic images of embedded software systems that do not have an explicit division into files. In this case, manually defining the Architecture will be extremely time-consuming, leading to many errors.

The specificity of destructive influences is determined by the scope of the Model—the CPDoSH, associated with the physical world. So, not only the elements of the Smart Home and its physical environment affect a person, but the physical space itself can destroy the media with the program code, thereby opening new vectors of violation of the integrity of the software. For example, a domestic cat in the house can spill a bowl of water, leading to a short circuit in the robot vacuum cleaner (the classic CPDoSH element), as a result of which an electrical breakdown will occur, which will partially destroy the MC in the device. The analysis of such code (both for finding out the causes of the incident and for tasks unrelated to the incident) will require the identification of the Architecture by the “surviving” sections of heterogeneous data.

Despite the fact that the total number of more or less popular Architectures (each of which can also be used in the CPDoSH), a specific Top-16 was chosen to build Signatures. This is due both to the presence of a specific assembly of the software product for the Architecture data (OS Gentoo), and to the fact that another set of Signatures was obtained by the authors in other researches. However, the proposed scheme of Prototype operation allows obtaining Signatures of practically any Architecture, and all that is required is the selection of a large product and its compilation for all the necessary types of Architectures.

In this article, a detailed description of the developed Prototype is omitted; it is also not fully tested. However, this Prototype is only an auxiliary element and is not directly related to the Model of MC. However, the architecture of the extended version of the Prototype, its algorithms and additional modes will be described in the continuation of the study. Extended testing of the Prototype will also be carried out, including checking its performance (and, consequently, the Model as a whole) under destructive effects on the MC.

We also note that the specifics of the CPDoSH area are taken into account in the work by considering a wide range of Architectures applicable for embedded systems (and not only classic Intel and AMD processors widely used in personal and server computers); taking into account the potential significant destruction of MC programs (without the possibility of their restoration by means of the OS); and the importance of investigating crimes involving physical harm to a person due to software vulnerabilities.

## 6. Conclusions

The conducted investigation was aimed at analyzing CPDoSH MC in the interests of information security.

This article is devoted to the CPDoSH MC Model, proposed for use in identifying the Architecture, operating in conditions when any information about this Architecture is absent in principle (for various reasons—deliberately changed, accidentally destroyed, known only at the time of execution, etc.).

The Model has an analytical form and takes into account the features of the structure and execution of programs. The advantage of the Model is its applicability for various tasks of MC research, and not only for architecture identification.

The proposed approach is based on the following Hypothesis—the byte-frequency signatures of MC of a given program can be used to unambiguously identify its Architecture.

Substantiation of the Hypothesis is identical to the Model adequacy and the Architecture identification efficiency.

In the course of the research, the formal Model of the CPDoSH MC was created, containing meta-information about its Architecture. A Hypothesis about the identification of the Architecture was put forward only by the frequency of occurrence of bytes encoding instructions of MC. An experiment with the developed Prototype made it possible to substantiate the validity of this Hypothesis. To construct the Signature, the Top-16 Architectures hypothetically used in the CPDoSH were taken.

Thus, a full cycle of research has been carried out: a theoretical model → advanced hypothesis → experimental proof.

The total size of all OS images for Top-16 Architectures was 3.2 GB. The total number of bytes for all 16 Architectures files for which Signatures were built (means only code segment without file header, data segment etc.) was $2.5\times 10^{9}$. Thus, on average, $\left(2.5\times 10^{9}\right)/16=1.5\times 10^{8}$ byte values were used for each of the Signatures. Therefore, near $\left(1.5\times 10^{8}\right)/256=6\times 10^{5}$ values were used to get the frequency of each byte. Such a sufficiently large number of values justifies the high accuracy of the Signatures.

Further development of the work should be the development of a full-fledged method for identifying the CPDoSH Architecture. This will be followed by the implementation of the method using a software tool, which can be based on the applied research prototype. We will also need to assess the consistency of the Model under various destructive effects on the CPDoSH MC. Interesting from a scientific and practical point of view will be the possibility of using the Model for programs inside CPDoSH that contain the MC of two or more Architectures.

## Figures and Tables

**Table 1:** Size of all executable files for each Top-16 Architecture for the CPDoSH.

No.	Architecture	Image File Name	Machine Code Size		ELF Header Identifier
			Bytes	Megabytes	
#1	Alpha	stage3-alpha-20200215T160133Z.tar.bz2	339.283.340	324	0x9026
#2	X32	stage3-x32-20210516T214503Z.tar.xz	222.718.092	212	0x3e
#3	Amd64	stage3-amd64-20210516T214503Z.tar.xz	203.487.272	194	0x3e
#4	Arm64	stage3-arm64-20210323T005051Z.tar.xz	179.237.608	171	0xb7
#5	Hppa64	stage3-hppa2.0-20200319T011207Z.tar.bz2	303.729.040	290	0xf
#6	I486	stage3-i486-20210517T214503Z.tar.xz	196.319.128	187	0x3
#7	I686	stage3-i686-20210517T214503Z.tar.xz	194.963.232	186	0x3
#8	Ia64	stage3-ia64-20210519T033521Z.tar.xz	224.309.636	214	0x32
#9	Mips	stage3-mips32r2-20140316.tar.bz2	164.983.958	157	0x8
#10	Mips64	stage3-mips64r2_multilib-20140904.tar.bz2	210.324.411	201	0x8
#11	Ppc	stage3-ppc-20210516T102000Z.tar.xz	187.421.256	179	0x14
#12	Ppc64	stage3-ppc64-20210516T102000Z.tar.xz	192.572.304	184	0x15
#13	RiscV64	stage3-rv64_lp64-20210509T171126Z.tar.xz	184.422.060	176	0xf3
#14	S390	stage3-s390-20200531T164023Z.tar.xz	189.763.308	181	0x16
#15	S390x	stage3-s390x-20200531T164536Z.tar.xz	191.553.620	183	0x16
#16	Sparc64	stage3-sparc64-20210420T135502Z.tar.xz	164.675.652	157	0x2b

For ease of reading, thousandths of MC sizes are separated by a dot.

**Table 2:** Comparative analysis of the proposed Model with existing analogues.

Analytical Modeling of MC	Criteria						Score
	A	B	C	D	E	F	
1. Malware detection by data mining techniques based on positionally dependent features [49]	+/−	+	+/−	+/−	+	+/−	4
2. Fine-Grained Compiler Identification With Sequence-Oriented Neural Modeling [50]	+	+	−	+	+	+/−	4.5
3. Function Identification in Android Binaries With Deep Learning [51]	−	+	−	+/−	+	+	3.5
4. Polymorphic Malware Detection Using Hierarchical Hidden Markov Model [52]	+/−	−	+	−	−	−	1.5
5. Feature Extraction Method for Cross-Architecture Binary Vulnerability Detection [53]	+	+/−	+/−	−	+/−	+/−	3
6. Vision-Based Malware Detection: A Transfer Learning Approach Using Optimal ECOC-SVM Configuration [13]	−	+	−	−	−	+/−	1.5
7. Cross-Architecture Intemet-of-Things Malware Detection Based on Graph Neural Network [54]	+	+	+/−	+	+/−	−	4
8. Asteria: Deep Learning-based AST-Encoding for Cross-platform Binary Code Similarity Detection [55]	+	−	+/−	+/−	+/−	−	2.5
Proposed Model	+	+	+	+	+	+	6

The following designations and points were used: “+”—full compliance with the criterion (1 point); “+/−”—partial compliance with the criterion (0.5 points); “−”—failure to meet the criterion. Criterion (0 points).

## Data Availability

Not applicable.

## References

[1] Zou Y., Zhu J., Wang X., Hanzo L. (2016). A survey on wireless security: Technical challenges, recent advances, and future trends. Proc. IEEE.

[2] Chen X., Makki K., Yen K., Pissinou N. (2009). Sensor network security: A survey. IEEE Commun. Surv. Tutor..

[3] Kim A., Oh J., Ryu J., Lee K. (2020). A Review of insider threat detection approaches with IoT perspective. IEEE Access.

[4] Kao D.Y., Wang S.J., Mathur K., Jain S., Huang F.F.Y. Privacy concealments: Detective strategies unveiling cyberstalking on Internet. Proceedings of the IEEE Asia-Pacific Services Computing Conference.

[5] Zdankin P., Weis T. Longevity of Smart Homes. Proceedings of the IEEE International Conference on Pervasive Computing and Communications Workshops (PerCom Workshops).

[6] Güven E.Y., ÇAMURCU A.Y. Physical attack detection for smart objects. Proceedings of the International Conference on Artificial Intelligence and Data Processing (IDAP).

[7] Fagbola F.I., Venter H.S. (2022). Smart digital forensic readiness model for shadow IoT devices. Appl. Sci..

[8] Tahtaci B., Canbay B. Android Malware Detection Using Machine Learning. Proceedings of the Innovations in Intelligent Systems and Applications Conference (ASYU).

[9] Filus K., Boryszko P., Domańska J., Siavvas M., Gelenbe E. (2021). Efficient feature selection for static analysis vulnerability Prediction. Sensors.

[10] Enders S., Rybalka M., Padilla E. PIdARCI: Using assembly instruction patterns to identify, annotate, and revert compiler idioms. Proceedings of the 18th International Conference on Privacy, Security and Trust (PST).

[11] Pereira J.D., Campos J.R., Vieira M. Machine learning to combine static analysis alerts with software metrics to detect security vulnerabilities: An empirical study. Proceedings of the 17th European Dependable Computing Conference (EDCC).

[12] Pizzolotto D., Inoue K. (2021). Identifying compiler and optimization level in binary code from multiple architectures. IEEE Access.

[13] Wong W.K., Juwono F.H., Apriono C. (2021). Vision-based malware detection: A transfer learning approach using optimal ECOC-SVM configuration. IEEE Access.

[14] Aslanyan H., Arutunian M., Keropyan G., Kurmangaleev S., Vardanyan V. BinSide: Static analysis framework for defects detection in binary code. Proceedings of the Ivannikov Memorial Workshop (IVMEM).

[15] Kotenko I., Saenko I., Skorik F., Bushuev S. Neural network approach to forecast the state of the Internet of Things elements. Proceedings of the XVIII International Conference on Soft Computing and Measurements (SCM).

[16] Izrailov K. (2021). The genetic decompilation concept of the telecommunication devices machine code. Proc. Telecommun. Univ..

[17] Clemens J. (2015). Automatic classification of object code using machine learning. Digit. Investig..

[18] Fernandes E., Jung J., Prakash A. Security analysis of emerging Smart Home applications. Proceedings of the IEEE Symposium on Security and Privacy (SP).

[19] Beckman B., Haile J. Binary analysis with architecture and code section detection using supervised machine learning. Proceedings of the IEEE Security and Privacy Workshops (SPW).

[20] Hu W., Chen T., Zhang N., Ma J. Adjust ELF format for multi-core architecture. Proceedings of the International Conference on Electronic Computer Technology.

[21] Atamaner M., Ergin O., Ottavi M., Reviriego P. Detecting errors in instructions with bloom filters. Proceedings of the IEEE International Symposium on Defect and Fault Tolerance in VLSI and Nanotechnology Systems (DFT).

[22] Ma Y., Han L., Ying H., Yang S., Zhao W., Shi Z. SVM-based instruction set identification for grid device firmware. Proceedings of the IEEE 8th Joint International Information Technology and Artificial Intelligence Conference (ITAIC).

[23] Ramljak M. Security analysis of open home automation bus system. Proceedings of the 40th International Convention on Information and Communication Technology, Electronics and Microelectronics (MIPRO).

[24] Wehmeyer L., Jain M., Steinke S., Marwedel P., Balakrishnan M. (2001). Analysis of the influence of register file size on energy consumption, code size, and execution time. IEEE Trans. Comput.-Aided Des. Integr. Circuits Syst..

[25] Zhao K., Bian J. Peeling algorithm for custom instruction identification. Proceedings of the IEEE Asia Pacific Conference on Circuits and Systems.

[26] Haaß M., Bauer L., Henkel J. (2014). Automatic custom instruction identification in memory streaming algorithms. Proceedings of the International Conference on Compilers, Architecture and Synthesis for Embedded Systems, CASES ’14.

[27] Zheng Y., Liu F., Yang C., Luo X., Zhao K. Identification of steganography software based on core instructions template matching. Proceedings of the Third International Conference on Multimedia Information Networking and Security.

[28] Radwan A.M. Machine learning techniques to detect maliciousness of portable executable files. Proceedings of the International Conference on Promising Electronic Technologies (ICPET).

[29] Shukla H., Patil S., Solanki D., Singh L., Swarnkar M., Thakkar H.K. On the design of supervised binary classifiers for malware detection using portable executable files. Proceedings of the IEEE 9th International Conference on Advanced Computing (IACC).

[30] Jophin S., Vijayan M., Dija S. Detecting forensically relevant information from PE executables. Proceedings of the International Conference on Recent Trends in Information Technology (ICRTIT).

[31] Kim Y., Moon J., Cho S.J., Park M., Han S. Efficient identification of Windows executable programs to prevent software piracy. Proceedings of the Eighth International Conference on Innovative Mobile and Internet Services in Ubiquitous Computing.

[32] Atluri V. Malware classification of portable executables using tree-based ensemble machine learning. Proceedings of the SoutheastCon.

[33] Al-Khshali H.H., Ilyas M., Ucan O.N. Effect of PE file header features on accuracy. Proceedings of the IEEE Symposium Series on Computational Intelligence (SSCI).

[34] Yousaf M.S., Durad M.H., Ismail M. Implementation of portable executable file analysis framework (PEFAF). Proceedings of the 16th International Bhurban Conference on Applied Sciences and Technology (IBCAST).

[35] AL-Nabhani Y., Zaidan A., Zaidan B., Jalab H.A., Alanazi H. A new system for hidden data within header space for EXE-file using object oriented technique. Proceedings of the 3rd International Conference on Computer Science and Information Technology.

[36] Zikratov I., Pantiukhin I., Krivtsova I., Druzhinin N. The method of ELF-files identification based on the metric classification algorithms. Proceedings of the 18th Conference of Open Innovations Association and Seminar on Information Security and Protection of Information Technology (FRUCT-ISPIT).

[37] Konaray S.K., Toprak A., Pek G.M., Akçekoce H., Kılınç D. Detecting file types using machine learning algorithms. Proceedings of the Innovations in Intelligent Systems and Applications Conference (ASYU).

[38] Garcia J. Duplications and misattributions of file fragment hashes in image and compressed files. Proceedings of the 9th IFIP International Conference on New Technologies, Mobility and Security (NTMS).

[39] Lee Y., Kwon H., Choi S.H., Lim S.H., Baek S.H., Park K.W. (2019). Instruction2vec: Efficient preprocessor of assembly code to detect software weakness with CNN. Appl. Sci..

[40] Bhatt M., Mishra A., Kabir M.W.U., Blake-Gatto S.E., Rajendra R., Hoque M.T., Ahmed I. (2020). Hierarchy-based file fragment classification. Mach. Learn. Knowl. Extr..

[41] Kwon Y.M., An J.J., Lim M.J., Cho S., Gal W.M. (2020). Malware classification using simhash encoding and PCA (MCSP). Symmetry.

[42] Yewale A., Singh M. Malware detection based on opcode frequency. Proceedings of the International Conference on Advanced Communication Control and Computing Technologies (ICACCCT).

[43] Bondarev S.E., Prokhorov A.S. Analysis of internal threats of the system “Smart Home” and assessment of ways to prevent them. Proceedings of the IEEE Conference of Russian Young Researchers in Electrical and Electronic Engineering (EIConRus).

[44] Yang J., Xie Y., Chen T. Research on web server application on multi-core embedded system. Proceedings of the International Conference on Embedded Software and Systems.

[45] Gao Y.X., Qi D.Y. Analyze and detect malicious code for compound document binary storage format. Proceedings of the International Conference on Machine Learning and Cybernetics.

[46] Zhao Z., Islam S., Hashemnia N., Hu D., Yao C. Understanding online frequency response signatures for transformer winding deformation: Axial displacement simulation. Proceedings of the International Conference on Condition Monitoring and Diagnosis (CMD).

[47] (2021). OS Gentoo Official Site. https://www.gentoo.org/.

[48] Izrailov K. (2021). Dataset of Files with Machine Code from Unpacked Gentoo OS Images for Various Processor Architectures. http://demono.ru/projects/MCArchIdent/SignaturesByGentoo/.

[49] Komashinskiy D., Kotenko I. Malware detection by data mining techniques based on positionally dependent features. Proceedings of the 18th Euromicro Conference on Parallel, Distributed and Network-Based Processing.

[50] Tian Z., Huang Y., Xie B., Chen Y., Chen L., Wu D. (2021). Fine-grained compiler identification with sequence-oriented neural modeling. IEEE Access.

[51] Sharif A., Nauman M. Function identification in Android binaries with deep learning. Proceedings of the Seventh International Symposium on Computing and Networking (CANDAR).

[52] Muhaya F.B., Khan M.K., Xiang Y. Polymorphic malware detection using hierarchical hidden Markov model. Proceedings of the IEEE Ninth International Conference on Dependable, Autonomic and Secure Computing.

[53] Li Z., Washizaki H., Fukazawa Y. Feature extraction method for cross-architecture binary vulnerability detection. Proceedings of the IEEE 10th Global Conference on Consumer Electronics (GCCE).

[54] Li C., Shen G., Sun W. Cross-architecture Intemet-of-Things malware detection based on graph neural network. Proceedings of the International Joint Conference on Neural Networks (IJCNN).

[55] Yang S., Cheng L., Zeng Y., Lang Z., Zhu H., Shi Z. Asteria: Deep learning-based AST-encoding for cross-platform binary code similarity detection. Proceedings of the 51st Annual IEEE/IFIP International Conference on Dependable Systems and Networks (DSN).

